# Low-Temperature Selective Growth of Tungsten Oxide Nanowires by Controlled Nanoscale Stress Induction

**DOI:** 10.1038/srep18265

**Published:** 2015-12-15

**Authors:** Hyungjoo Na, Youngkee Eun, Min-Ook Kim, Jungwook Choi, Jongbaeg Kim

**Affiliations:** 1 School of Mechanical Engineering, Yonsei University, 50 Yonsei-ro, Seodaemun-gu, Seoul 120-749, Republic of Korea

## Abstract

We report a unique approach for the patterned growth of single-crystalline tungsten oxide (WO_x_) nanowires based on localized stress-induction. Ions implanted into the desired growth area of WO_x_ thin films lead to a local increase in the compressive stress, leading to the growth of nanowire at lower temperatures (600 °C vs. 750–900 °C) than for equivalent non-implanted samples. Nanowires were successfully grown on the microscale patterns using wafer-level ion implantation and on the nanometer scale patterns using a focused ion beam (FIB). Experimental results show that nanowire growth is influenced by a number of factors including the dose of the implanted ions and their atomic radius. The implanted-ion-assisted, stress-induced method proposed here for the patterned growth of WO_x_ nanowires is simpler than alternative approaches and enhances the compatibility of the process by reducing the growth temperature.

The wide direct band gap of tungsten oxide (WO_x_)^1^ makes it an attractive material for photoelectrochemical devices^2^,^3^ and gas sensors^4^. The high surface to volume ratio and photon absorption efficiency of WO_x_ nanowires^5^ can be used to improve the performances of these devices, in particular the saturation photocurrent, the sensitivity, and the threshold field respectively of photoelectrochromical devices^6^,^7^, gas sensors^8^, and field-emission displays^9^.

WO_x_ nanowires are typically grown by vapor-solid (VS)^10^,^11^,^12^,^13^,^14^,^15^, vapor-liquid-solid (VLS) synthesis^16^,^17^, or hydrothermal synthesis^18^,^19^. In VS synthesis, a WO_x_ layer is formed on a tungsten wire, tip, or filament, on which WO_x_ nanowires grow under high-temperature annealing in an oxygen environment. Aligned nanowires can be grown using a KI catalyst by VLS synthesis at around 600–650 °C whereas hydrothermal synthesis is the preferred method for the large-scale production of WO_x_ nanowires. Klinke *et al.*^20^. have developed an alternative method whereby WO_x_ nanowires are grown by annealing WO_3_/W substrates under hydrocarbon gas. The authors explain that in this case, the nanowires are the result of interfacial strain-induced carbide formation between the WO_3_ and W layers. Elsewhere, the patterned growth of WO_x_ nanowires has been demonstrated by Nakao and colleagues^21^,^22^,^23^; the WO_x_ nanowires synthesized by annealing W/Cr thin films on Si substrates in a vacuum furnace with flowing oxygen gas are arranged into patterns using a micro heater or lift-off techniques. In this approach the nanowires extend via solid-phase growth from nanowire nuclei generated on the uneven W surface. To date however, although controlling the site and density of nanowires is a crucial factor for the practical application of nanowire devices, the growth of WO_x_ nanowires in nanoscale patterns has seldom been investigated.

In our work, the driving force for the growth of nanowires is compressive stress induced from the difference in thermal expansion coefficients (TECs) and ion implantation. Compared to other growth methods of WO_x_ nanowires such as VS-mechanism^10^,^11^,^12^,^13^,^14^,^15^, a major benefit of our approach is high process compatibility due to low growth temperature. Moreover, this growth method does not require any precursor or catalyst.

In this study, by implanting ions into WO_x_ thin films deposited on Si substrates, we have successfully synthesized WO_x_ nanowires at 600 °C, 150 °C lower than for non-implanted films. This approach is based on stress-induced growth, whereby the compressive stress applied to thin films is relieved by the growth of nanowires. Compressive stress can be applied by oxidation-induced volume expansion^24^,^25^, external loading^26^, or by exploiting the difference between the TECs of the thin film and the substrate^27^,^28^. Here, the implanted ions generate additional compressive stress inside the film allowing nanowires to nucleate at lower temperatures (viz. 600 °C instead of 750 °C). The difference in the minimum nanowire growth temperature between the ion-implanted and non-implanted regions ensures that the patterns are grown in a site-specific manner, both on the micro- and nanoscale. Indeed, a single nanowire was successfully grown by reducing the ion-implanted area down to 50 × 50 nm^2^, and wafer-scale growth of patterned nanowires is also possible. This is the first time the growth of WO_x_ nanowires has been controlled on the nanoscale using localized compressive stress. Furthermore, this report explains the mechanisms governing the growth of WO_x_ nanowires induced by the difference between the TECs of the thin film and the substrate.

## Results

### Stress-induced growth at lower temperature

The nanowires were grown using a conventional two-step process. First, a 10-nm-thick WO_x_ film was deposited on a (100) Si substrate by electron-beam evaporation using 99.9% WO_3_ powder as an evaporation source. The substrate was then loaded into a furnace and annealed at temperatures above 700 °C for 20 min in a low-pressure environment with 100 sccm flowing N_2_ (ca. 11 Torr). Supplementary Fig. 1 shows scanning and transmission electron microscopy (SEM and TEM) images of WO_x_ nanowires synthesized without ion implantation at 700–950 °C. The high resolution TEM (HR-TEM) images of the nanowires grown at 750 °C are shown in Supplementary Fig. 1g, h. The length and density of the nanowires increases for growth temperatures up to 800 °C (Supplementary Fig. 1a–c), at which the nanowire density is maximal. On increasing the temperature to 900 °C the nanowires become more elongated but are less densely packed (Supplementary Fig. 1c–e). Supplementary Fig. 1f shows that no nanowires grow above 950 °C due to the crystallization of the thin film. Between 750 °C and 900 °C, the length of the nanowires is proportional to the growth temperature which in turn is directly related to the compressive stress. The selective-area electron diffraction (SAED) pattern (Supplementary Fig. 1g inset) highlights the crystallinity and the growth direction of the nanowires. Supplementary Fig. 1h shows that the nanowire is single crystalline with monoclinic WO_x_ grown in [010] direction. Lattice constants of two planes, a = 0.377 nm and b = 0.467 nm, agree with a = 0.378 {010} and b = 0.459 {103} according to JCPDS Card No. 36–101.

These results confirm that it is the stress induced by the difference (9.6 × 10^−6^ m/mK^−1^)^29^ between the TECs of the WO_x_ thin film (12 × 10^−6^ m/mK^−1^) and the Si substrate (2.4 × 10^−6^ m/mK^−1^) that leads to the growth of these nanowires. (Note that growth by oxidation-induced volume expansion of the metallic film, mentioned above, is not possible in the oxygen-less low-pressure N_2_ environment used here.) These observations lead us further to the investigation of ion implantation as a means to increase the compressive stress in the thin film and grow nanowires at relatively low temperatures.

**Figure 1:**
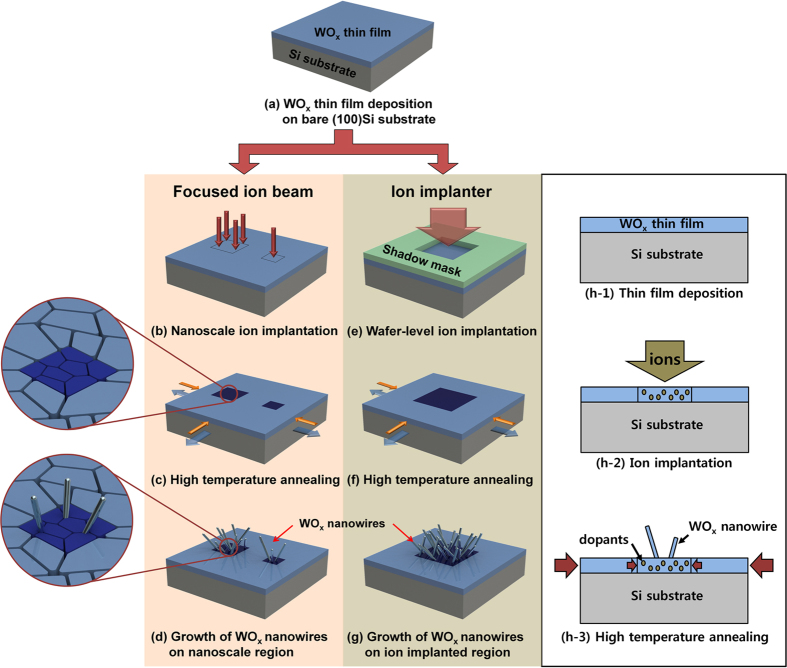
Schematics of the different steps involved in the site-selective growth of WO_x_ nanowires using the local stress induced by implanted ions. (**a**) A WO_x_ thin film is deposited on a (100) Si substrate by electron-beam evaporation. (**b**–**d**) Processing using a focused ion beam; (**b**) Ga ions are implanted in nanoscale areas; (**c**) the sample is annealed at 600 °C; (**d**) a small number of WO_x_ nanowires grow in the Ga-ion-implanted nanoscale region. The magnified schematic depictions of the regions highlighted in (**c**,**d**) show the nanoscale morphology of the WO_x_ thin films and nanowires in the ion-implanted (dark blue) regions where the grains are smaller. (**e**–**g**) Processing using an ion implanter; (**e**) As, Ar, or N_2_ dopants are selectively implanted through a shadow mask onto the WO_x_ thin film; (**f**) compressive stress arises from the difference between the thermal expansion coefficients of the thin film and the substrate during annealing at 600 °C in a furnace as in (**c**,**g**) WO_x_ nanowires grow only in the ion-implanted region because the ions increase the compressive stress locally. (h1-3) Cross-sectional diagram of growth mechanism using compressive stress induced from the difference in TECs and ion implantation. Large red arrows indicate the compressive stress from difference in TECs; small red arrows indicate the additional compressive stress from ion implantation in the desired area. (h1-3) Cross-sectional diagram showing the growth of nanowires due to compressive stress. Large red arrows indicate the compressive stress from difference in TECs; small red arrows indicate the additional compressive stress from ion implantation in the desired area.

Figure 1 outlines the three steps involved in the implanted-ion-assisted, stress-induced method developed here. First, a 10-nm-thick WO_x_ film is deposited on a bare Si wafer by electron-beam evaporation using 99.9% WO_3_ powder as an evaporation source (Fig. 1a). We tested Ga, As, Ar, and N_2_ ions to induce additional stress at reduced temperatures. For patterned growth characterization, As and Ga were used for ion implanter and focused ion beam, respectively. Ion implantation step was performed without heating the substrate. Figure 1b–d illustrates the similar steps with Ga ions implanted by a focused ion beam (FIB) into WO_x_ films for nanoscale patterning. After ion implantation, the substrate is heated to increase the stress levels (Fig. 1c), and the WO_x_ nanowires are synthesized on the ion-implanted nanoscale patterns (Fig. 1d). Figure 1e–g shows the patterned ion injection steps in the ion implanter using As, Ar, N_2_ ions. Again, after implanting ions in selected areas using a shadow mask (Fig. 1e), the substrate is annealed (Fig. 1f). Compressive stress is induced from the difference in TECs and also from the implanted ions such that the threshold stress for the growth of WO_x_ nanowires is reached in the ion-implanted region at a lower temperature than in the non-implanted surroundings (Fig. 1g). All the heating steps were conducted in a N_2_ environment (ca. 11 Torr) at 600 °C for 60 min.

### Effect of dose and patterned growth

**Figure 2:**
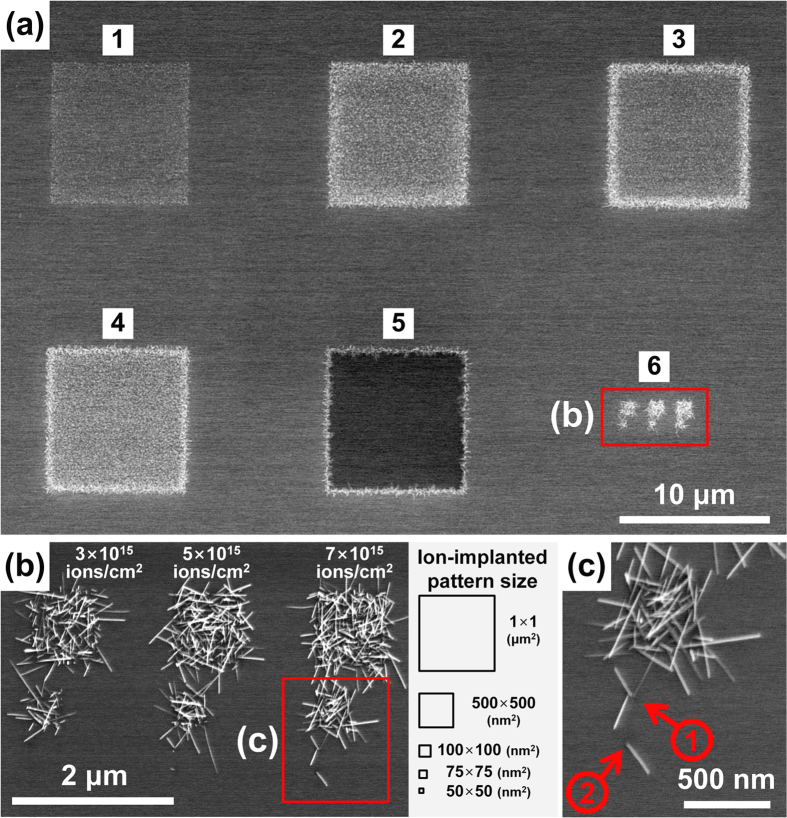
Scanning electron micrographs of WO_x_ nanowires grown in the Ga-ion-implanted regions of WO_x_ thin films. The samples were annealed at 600 °C for 60 min in a low-pressure environment under N_2_ flow. (**a**) Square 10 × 10 μm^2^ patterns numbered 1 to 5 implanted using a 30 keV focused ion beam (FIB) with Ga doses of 1 × 10^15^, 3 × 10^15^, 5 × 10^15^, 7 × 10^15^, and 9 × 10^15^ ions/cm^2^, respectively. (**b**) Region number 6 in (a) contains three nanoscale patterns obtained with doses (from left to right) of 3 × 10^15^, 5 × 10^15^, and 7 × 10^15^ ions/cm^2^ implanted using a FIB in areas (from top to bottom) of 1000 × 1000, 500 × 500, 100 × 100, 75 × 75, and 50 × 50 nm^2^, as depicted on the right of the panel. (**c**) High-magnification images of the region within the red rectangle in (**b**). The red arrows numbered 1 and 2 indicate the nanowires in the 100 × 100 and 75 × 75 nm^2^ squares, respectively.

Figure 2 shows SEM images of the WO_x_ nanowires synthesized on WO_x_ film using various doses of Ga ions implanted at 30 keV by FIB and following annealing at 600 °C for 60 min. The square patterns numbered 1–5 in Fig. 2a are 10 × 10 μm^2^ in area and have implanted Ga doses of 1 × 10^15^, 3 × 10^15^, 5 × 10^15^, 7 × 10^15^, and 9 × 10^15^ ions/cm^2^, respectively. These results highlight the optimal ion dose to maximize the number density and length of the nanowires grown. The nanowires in pattern number 1 are short and relatively sparsely packed because the dose of 1 × 10^15^ ions/cm^2^ is insufficient to provide the compressive stress necessary to initiate growth. There are no nanowires in pattern number 5 (9 × 10^15^ ions/cm^2^) because the WO_x_ film becomes etched at higher doses. Long nanowires (>400 nm) are visible in patterns 2, 3, and 4 (3 × 10^15^, 5 × 10^15^, and 7 × 10^15^ ions/cm^2^, respectively).

For nanoscale patterning, doses of 3 × 10^15^, 5 × 10^15^, and 7 × 10^15^ ions/cm^2^ where chosen, as shown in Fig. 2b (an enlarged view of pattern number 6 in Fig. 2a). Figure 2c shows a high magnification image of the nanoscale pattern obtained with a dose of 7 × 10^15^ ions/cm^2^, and corresponds to the region within the red square in Fig. 2b. The three sets of patterns in Fig. 2b are each composed of nanowires grown on five square areas covering (from top to bottom) 1000 × 1000, 500 × 500, 100 × 100, 75 × 75, and 50 × 50 nm^2^. The nanowires are 10–20 nm in diameter and 300–1000 nm in length. The arrows numbered 1 and 2 in Fig. 2c respectively point out the three nanowires grown on the 100 × 100 nm^2^ square and the single nanowire grown on the 75 × 75 nm^2^ square. No nanowires are visible in Fig. 2c for the 50 × 50 nm^2^ square; on the other hand, a single nanowire was successfully synthesized with this pattern size by annealing at 630 °C for 20 min, as shown in Supplementary Fig. 2.

**Figure 3:**
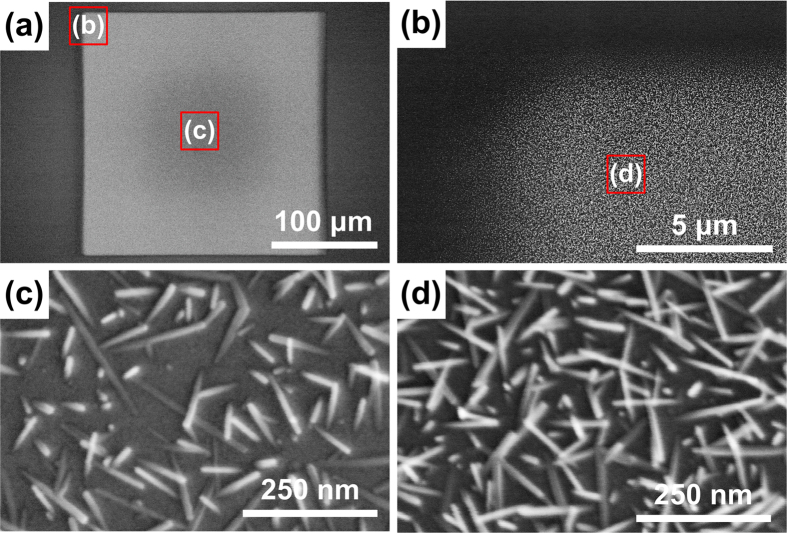
Scanning electron micrographs of WO_x_ nanowires grown on As-ion-implanted (5 × 10^15^ ions/cm^2^) patterns obtained using an ion implanter operating at 30 keV. The samples were then annealed at 600 °C for 60 min. (**a**) Patterned growth of WO_x_ nanowires using a shadow mask, with (**b**) a magnified view of the top left corner of the square. High-resolution images showing nanowires grown (**c**) in the center and (**d**) in the corner of the pattern.

Wafer-scale patterned growth of the WO_x_ nanowires was realized using an ion implanter. As shown in Fig. 1b–d, the ions were implanted through a shadow mask placed on a 4 inch (10.16 cm) Si wafer coated with a 10-nm-thick WO_x_ thin film. Figure 3 shows the nanowires grown after implanting As at 5 × 10^15^ ions/cm^2^ and 30 keV. Figure 3b shows an enlarged view of the corner of the square pattern shown in Fig. 3a, while Fig. 3c,d respectively show high magnification images of the center and the corner of the pattern.

### Effect of dopants

**Figure 4:**
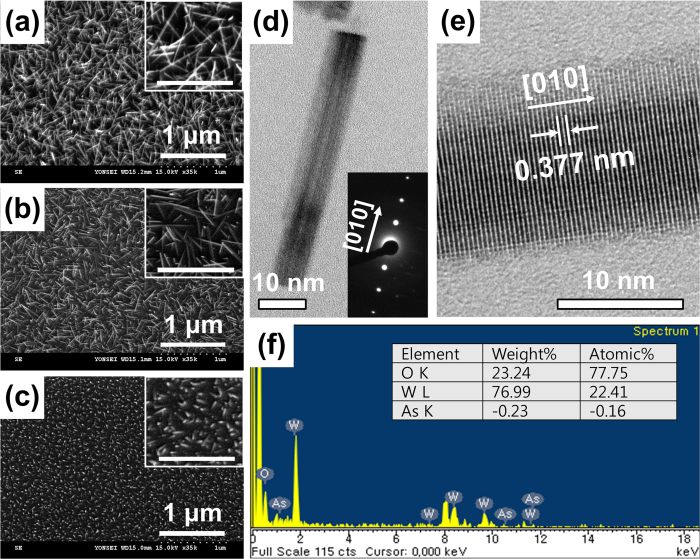
WO_x_ nanowires grown using an ion implanter with As, Ar, and N_2_ dopants. All the samples were annealed at 600 °C for 60 min in a low-pressure environment under N_2_ flow. Scanning electron micrographs of the WO_x_ nanowires grown on (**a**) As-, (**b**) Ar-, and (**c**) N_2_-ion-implanted thin films. All dopants were implanted at 30 keV at a dose of 5 × 10^15^ ions/cm^2^. The insets in (**a**–**c**) show the corresponding high-magnification (scale bar: 500 nm) images. (**d**) A high-resolution scanning electron micrograph of a WO_x_ nanowire grown on the As-ion-implanted sample shown in (**a**), with (inset) the corresponding selected area diffraction pattern. (**e**) An expanded view of the same nanowire clearly showing the lattice spacing and its axial direction. (**f**) Energy-dispersive X-ray spectrum showing the elemental content of the WO_x_ nanowires grown on the As-implanted sample shown in (**a**).

Because compressive stress plays a major role in the synthesis of these nanowires, implanting larger ions should facilitate nanowire growth. To investigate this effect, three bare Si wafers were coated with identical 10-nm-thick WO_x_ films by electron-beam evaporation and different ions, As, Ar, and N_2_ were implanted onto each of three wafers at 5 × 10^15^ ions/cm^2^ and 30 keV by an ion implanter. Figure 4a–c show SEM images, with magnified views inset, of the WO_x_ nanowires—respectively 300–500 nm, 100–200 nm and <100 nm long—synthesized on the three different wafers after annealing at 600 °C for 60 min.

Figure 4d shows an HR-TEM image and the corresponding SAED pattern obtained from one of the WO_x_ nanowires shown in Fig. 4a, grown by As implantation. As described above for the non-implanted results (Supplementary Fig. 1), the nanowire has a single-crystalline structure. The SAED patterns in Fig. 4d highlight growth in the (010) direction and the spacing of the lattice planes is visible in the magnified image (Fig. 4e). The energy dispersive X-ray spectrum in Fig. 4f reveals that the nanowire consists of 78% oxygen and 22% tungsten. The fact that the nanowire contains As less than the error range demonstrates that the implanted ions increase the compressive stress locally in the thin film without doping the resulting nanowires.

## Discussion

### Growth mechanism

Nanowire growth mechanism used in our research is based on atomic diffusion enhanced by compressive stress in thin film from the difference in thermal expansion coefficient (TECs) between thin film and the substrate at high annealing temperature^27^,^28^. In this stress-induced method, WO_x_ thin film has larger TECs than Si substrate (TEC of WO_x_: 12 × 10^−6^ m/mK^−1^, TEC of Si: 2.4 × 10^−6^ m/mK^−1^) and is impeded to expand by the substrate, inducing compressive stress in it. In order to release the stress, nanowires are grown on the surface of the thin film by atomic diffusion at grain boundaries^27^,^28^.

It is widely known that the implanted dopants also generate the compressive stress inside the target^30^,^31^. In our work, we utilized the compressive stress induced by both thermal mismatch and ion implantation for low temperature selective growth of WO_x_ nanowires. As shown in Fig. 1(h), differences in TECs makes the entire thin film to be subjected to compressive stress, and implanted dopants generate additional compressive stress to facilitate the growth of nanowires only on the desired regions where ions are implanted. The magnitude of the compressive stress is proportional to annealing temperature, and there exist a threshold stress level and the corresponding temperature that initiates the growth of nanowires. In our experimental condition, the threshold temperature to produce sufficient compressive stress for nanowire growth is 750 °C without ion implantation, as shown in Supplementary Fig. 1. Thus at 600 °C, the stress level at non-implanted area is below the threshold stress and no nanowire is grown, while the ion-implanted region gets additional compressive stress from the implanted ions, which make the total stress level higher than the threshold stress for nanowire growth. Resultantly, WO_x_ nanowires are grown only on the ion implanted area at a temperature lower than the threshold, 750 °C, as shown in the SEM images of Fig. 2.

As described previously^27^,^28^, grain boundaries act as nucleation sites for nanowire growth; however, nanowires did not grow at all the grain boundaries in the ion-implanted area. It seems that the number of activated nucleation sites is somewhat sensitively dependent on the annealing temperature. Comparing the identical patterned area of 100 × 100 nm^2^, when the annealing temperature is 600 °C, only 3 nanowires were grown (Fig. 2c), while 6 nanowires were observed at the annealing temperature of 630 °C (Supplementary Fig. 2b). To grow a single nanowire therefore, the annealing temperature and the implantation area and dose must all be carefully adjusted. Note furthermore that since the synthesis step was conducted at 600 °C, far below the melting point of W and WO_x_, the VS and VLS mechanisms are clearly not involved in the growth of these nanowires.

Ion implantation facilitates the growth of nanowires through two different effects, thereby reducing the temperature required for their synthesis. Firstly, the volume of the implanted ions increases the compressive stress in the implanted region of the thin films^30^,^31^. Secondly, the ion implantation leads to the formation of smaller grains in the thin films during annealing, thereby reducing the activation energy for atomic diffusion at the grain boundaries^27^,^28^. During annealing, the grains in the as-deposited WO_x_ thin films increase in size^32^; however, grain growth is hindered in the ion-implanted regions due to Zener pinning^33^. The relatively high surface-to-volume ratio of these smaller grains leads to a reduced activation energy for atomic diffusion^34^, and as a result, nanowires grow in the ion-implanted regions at relatively low temperatures (see Fig. 1f,g).

### Effect of stress magnitude

The increase in compressive stress in the films arising from the implanted ions is influenced by both the volume and the concentration of the implanted ions. The atomic/molecular radii of As, Ar, and N_2_ dopants are 0.121, 0.096 and 0.07/0.109 nm (short/long), respectively^35^, and we estimated their concentrations from the depth profiles measured by time-of-flight secondary ion mass spectrometry (TOF-SIMS) as shown in Supplementary Fig. 3. The projection ranges of As, Ar, and N_2_ were 8 nm, 10 nm, and 7.8 nm, respectively, such that the peak concentration of the ions was formed within the thickness of the WO_x_ thin film. However, since the doses were near the detection limit of the mass spectrometry, the concentrations of the implanted ions inside the thin film could not be measured accurately. For Ar and N_2_, which are less readily ionized, the measurements could have been further impaired by these ions becoming trapped inside the film rather than forming chemical bonds with tungsten and oxygen atoms. With identical implantation dose and energy, the growth results varied depending on the ion species injected. Hence it could be concluded that heavier and larger dopants increase the compressive stress and lead to the growth of longer nanowires.

The nanowires in Figs 4a and 2b are of a similar length because the atomic radii of As (0.121 nm) and Ga (0.124 nm) are almost identical. The nanowires in Fig. 4c (N_2_ implantation at 5 × 10^15^ ions/cm^2^) and Fig. 2a (Ga implantation at 1 × 10^15^ ions/cm^2^) are shorter because of the relatively low compressive stress arising respectively from the smaller atomic volume and the lower dose. These results show that the proposed method allows nanowires to be differently grown in length using different atomic sizes of various dopants. Additionally, the results obtained using Ar and N_2_ dopants imply that the implanted ions have no catalytic effect on the growth of nanowires. In our test, not only the metallic dopants (Ga) and the semi-metallic dopants (As) but also the gaseous dopants (Ar and N_2_) show similar results on the growth of nanowires when the growth temperature, pressure, and annealing time are identical. Therefore, it is unlikely for WO_x_ to nucleate by dopant catalysts.

In conclusion, we have successfully realized the scalable, site-specific growth of WO_x_ nanowires at relatively low temperatures by exploiting the localized stress induced by ions implanted into WO_x_ films. Experimental results show that the dopants increase the compressive stress locally, and that both their volume and dose have a direct impact on nanowire growth. That is, dopants with larger atomic radii lead to greater increases in stress, and thus to the growth of longer and more densely packed nanowires. Furthermore, since the extent of nanowire growth is governed by the size of the implantation area, reducing this down to the nanoscale allows a few or even a single isolated nanowire to be grown. The WO_x_ nanowires synthesized in this way are single crystalline in structure, and the proposed stress-induced method does not require any precursor or catalyst. The reduction in the growth temperature achieved by the additional stress as well as the scalability of the process could render WO_x_ nanowires amenable to a wider range of applications.

## Methods

### Thin Film Deposition

For the WO_x_ thin film deposition, 99.9% WO_3_ powder was used as an evaporation source and (100) Si wafer was used as the substrate material. 10-nm-thick WO_x_ film was electron-beam evaporated on to the Si wafer with a deposition rate of 0.5 Å/s.

### Ion Implantation

(1) Using the FIB: Ga ions were implanted into WO_x_ film with programmed patterns. The acceleration voltage, beam condition, and the beam size was 30 keV, 14 pA, and 14 nm, respectively. (2) Using the ion implanter: As, Ar, and N_2_ dopants were implanted into the WO_x_ film with an acceleration voltage of 30 keV.

### Annealing

The specimens were heated in a furnace (Lindberg Blue M TF55030C) at low-pressure N_2_ environment (ca. 11 Torr). Annealing temperature and time of un-implanted and implanted specimens were 700–900 °C for 20 min and 600 °C for 60 min, respectively.

## Additional Information

**How to cite this article**: Na, H. *et al.* Low-Temperature Selective Growth of Tungsten Oxide Nanowires by Controlled Nanoscale Stress Induction. *Sci. Rep.* **5**, 18265; doi: 10.1038/srep18265 (2015).

## Supplementary Material

Supplementary Information
