# Supplementary material for: Low-Temperature Selective Growth of Tungsten Oxide Nanowires by Controlled Nanoscale Stress Induction
Source: Sci Rep. 2015 Dec 15;5:18265. doi: 10.1038/srep18265 (PMC4678864; doi:10.1038/srep18265)
Supplement: Supplementary Information [file srep18265-s1.doc]

Low-Temperature Selective Growth of Tungsten Oxide Nanowires by Controlled Nanoscale Stress Induction

Hyungjoo Na, Youngkee Eun, Min-Ook Kim, Jungwook Choi, and Jongbaeg Kim*

School of Mechanical Engineering, Yonsei University, 50 Yonsei-ro, Seodaemun-gu, Seoul 120-749, Republic of Korea

1. **Growth of WO_x_ nanowires without ion implantation**





**2 μm**

**2 μm**

**50 nm**

**5 nm**

**Supplementary Figure 1.** Scanning electron micrographs of WO_x_ nanowires grown without ion implantation on (100) Si substrates at (a) 700°C, (b) 750°C, (c) 800°C, (d) 850°C, (e) 900°C, and (f) 950°C for 20 min in a low-pressure environment with 100 sccm flowing N_2_ (ca. 11 Torr). (g) Transmission electron micrograph of one of the nanowires in (b), with the corresponding selected area diffraction pattern inset. (h) High-resolution transmission electron micrograph of the nanowire in (g).

**(2) Nanoscale patterned growth of WO_x_ nanowires using a focused ion beam**

Supplementary Figure 2 shows the WO_x_ nanowires grown using the same process as for those shown in Fig. 2—that is, with Ga ions implanted at 5 × 10^15^ ions/cm^2^ and 30 keV—but with the samples annealed at 630°C for 20 min (rather than at 600°C for 60 min). Supplementary Figure 2a shows the nanoscale patterns arranged as described for Fig. 2b. Supplementary Figure 2b shows six, four, and a single nanowire respectively in the 100 × 100, 75 × 75, and 50 × 50 nm^2^ squares of the right-most sample in Supplementary Figure 2a. The red arrows numbered 1, 2, and 3 point towards the nanowires grown on the 100 × 100, 75 × 75, and 50 × 50 nm^2^ squares, respectively.


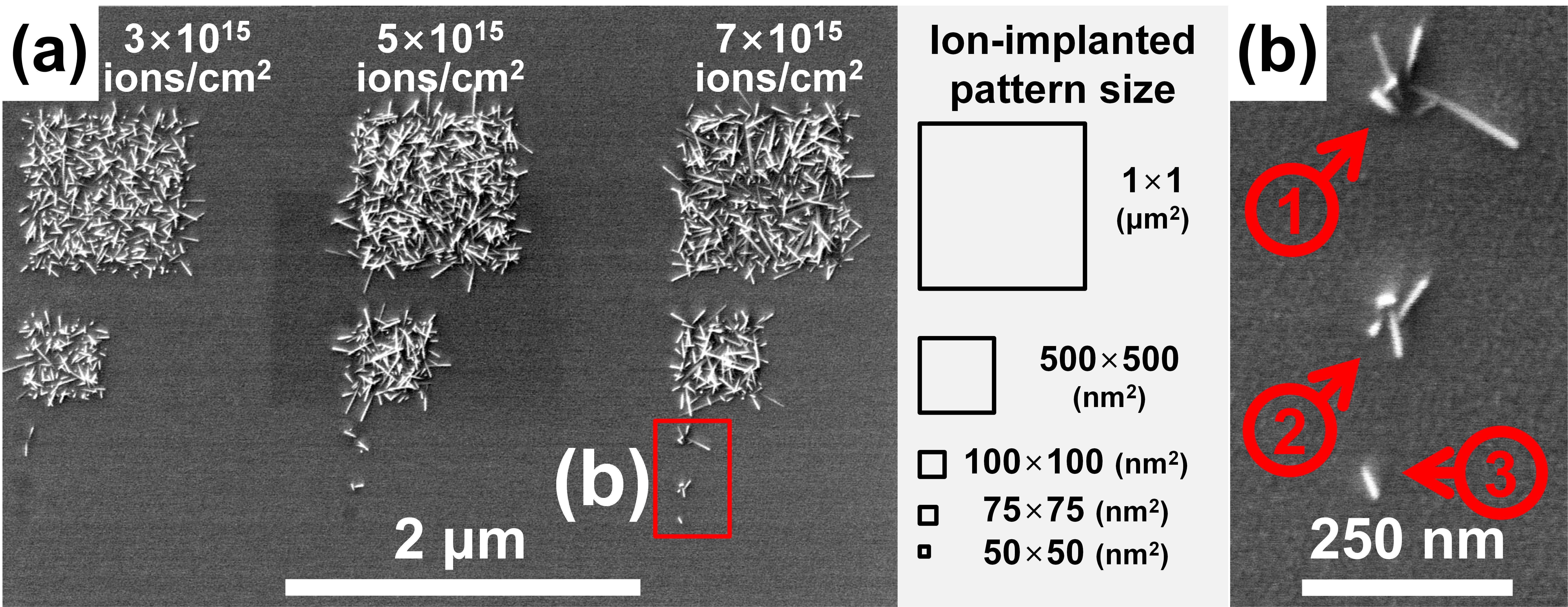


**Supplementary Figure 2.** Scanning electron micrographs showing WO_x_ nanowires grown on squares implanted using a 30 keV focused beam of Ga ions. The samples were annealed at 630°C for 20 min. (a) Images of the nanowire patterns obtained with Ga doses of (from left to right) 3 × 10^15^, 5 × 10^15^, and 7 × 10^15^ ions/cm^2^ on square patterns 1000 × 1000, 500 × 500, 100 × 100, 75 × 75, and 50 × 50 nm^2^ (from top to bottom) in area. (b) High-magnification image of the region within the red rectangle in (a). The red arrows numbered 1 to 3 indicate the nanowires in the 100 × 100, 75 × 75, and 50 × 50 nm^2^ squares, respectively.

**(3) Time-of-flight secondary ion mass spectrometry**

Supplementary Figure 3 shows the depth profile of As, Ar, and N implanted into WO_x_ thin films on the Si substrate before annealing.


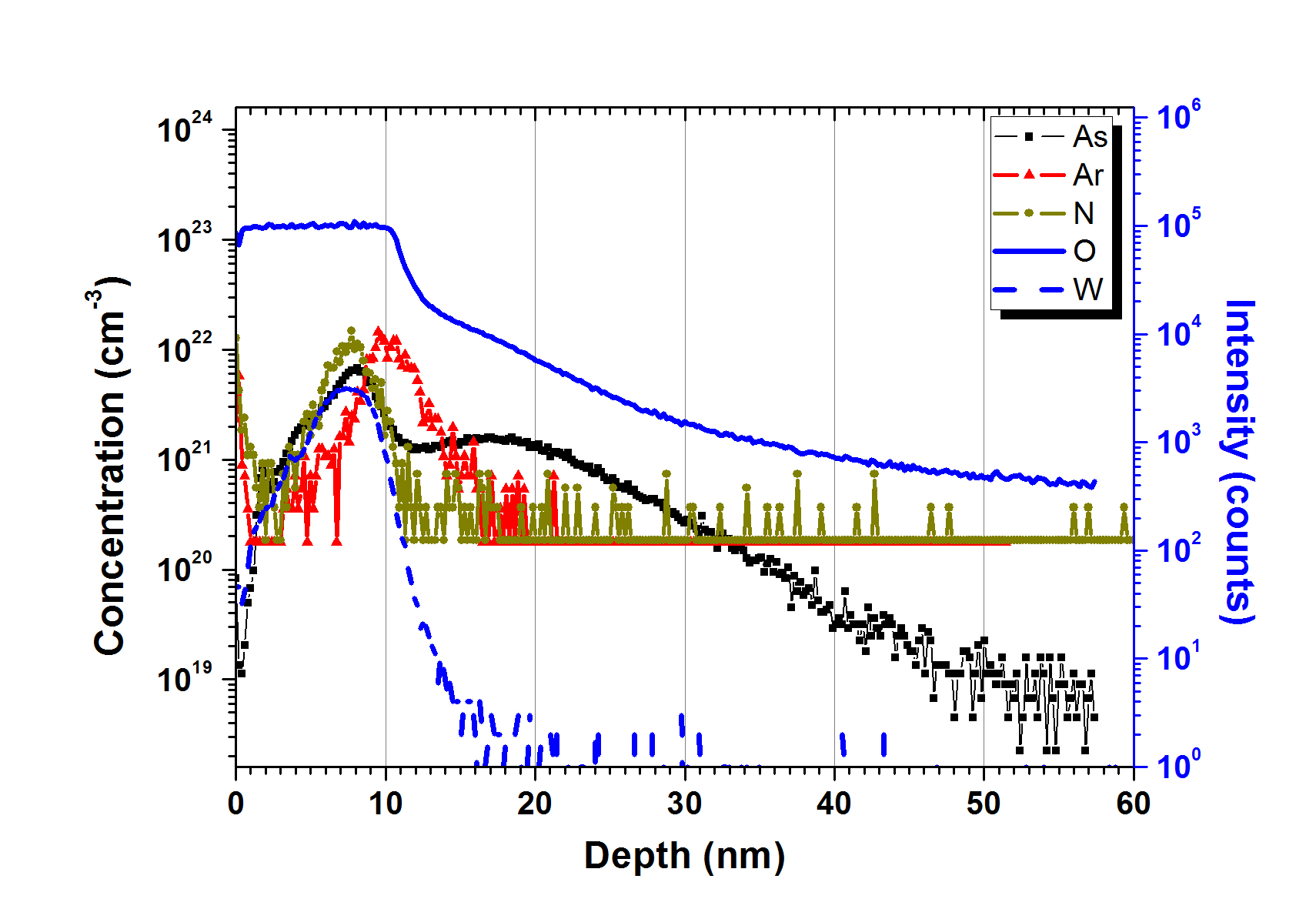


**Supplementary Figure 3.** Concentration profiles of As, Ar, N in WO_x_ thin films as measured by time-of-flight secondary ion mass spectrometry.
